# PDRNet: A Novel Physical Feature-Driven Residual Network for Motor Vibration Signal Denoising

**DOI:** 10.3390/s25237213

**Published:** 2025-11-26

**Authors:** Kaijie Yu, Xiongying Wu, Meng Yang, Fanqiang Lin, Zhuobang Liang

**Affiliations:** 1 College of Mechanical and Electrical Engineering, Chengdu University of Technology, Chengdu 610059, China; 202321020423@stu.cdut.edu.cn (K.Y.); linfq@cdut.edu.cn (F.L.); 2 School of Computer Science and Engineering, University of Electronic Science and Technology of China, Chengdu 610059, China; 202521080903@std.uestc.edu.cn; 3 College of Computer Science and Cyber Security, Chengdu University of Technology, Chengdu 610059, China; 202221020218@stu.cdut.edu.cn

**Keywords:** vibration signal denoising, physics-driven, deep learning, manifold learning

## Abstract

Bearing signal denoising is a pivotal task in predictive maintenance and condition monitoring of industrial machinery. However, conventional denoising methods often face difficulties in simultaneously suppressing noise and preserving essential physical features. To address this challenge, we propose a novel denoising framework that incorporates physical feature priors derived from low-dimensional manifold-based simulated data. Specifically, a regression branch—constructed using convolutional and residual neural networks—is integrated into the main denoising model to exploit the intrinsic structure of bearing signals. By embedding manifold-informed priors, the regression branch enhances denoising performance and ensures the retention of critical physical features. Experimental results demonstrate that the proposed approach surpasses traditional methods in both signal denoising and bearing fault diagnosis. Notably, the incorporation of manifold-derived priors improves the model’s capability to capture the underlying physical characteristics of the signals, indicating that the network has learned their inherent features rather than simply minimizing the loss function. Overall, this study introduces a robust denoising paradigm for complex industrial environments where bearing signals exhibit significant variability.

## 1. Introduction

Rolling bearings are among the most critical components in rotating machinery, and their health condition directly determines system safety, reliability, and efficiency. Undetected faults not only degrade performance but may also trigger unexpected shutdowns or even catastrophic accidents. Consequently, accurate monitoring and timely bearing fault diagnosis have long been central issues in mechanical engineering and intelligent maintenance.

In practice, vibration signals are the most widely used indicators for bearing fault diagnosis [1]. However, they are typically collected under complex operating conditions and are unavoidably contaminated by strong background noise [2,3]. Such noise can readily obscure weak but diagnostically significant features: periodic impacts, resonance responses, and modulation effects, thereby complicating a reliable diagnosis. Over the past few decades, widely adopted approaches, including wavelet transform (WT) [4,5,6], empirical mode decomposition (EMD) [7], and singular value decomposition (SVD) [8,9], have enabled multiscale time–frequency analysis and shown utility for nonstationary motor vibration signals. Nevertheless, time–frequency methods generally require careful choices of basis functions, decomposition scales, and thresholds, and their computational burden can be substantial. Suboptimal parameterization often compromises denoising performance in the presence of complex, heterogeneous noise and typically demands considerable engineering experience. As a result, conventional techniques, such as filtering and wavelet thresholding, may suppress noise to some extent, but frequently attenuate or distort fault-related information, leading to unstable and unreliable diagnostic results [10].

Recent years have witnessed growing interest in deep learning for bearing-signal denoising and fault diagnosis, owing to its superior capacity for nonlinear modeling and hierarchical feature extraction [11,12,13,14,15]. Some methods involve improving the fault detection model to adapt to the motor signals in noisy environments [16,17]. Yet most existing methods are purely data-driven and lack explicit characterization of the signal’s physical structure [18]. In the absence of physics-based constraints, models may overfit noise patterns or inadvertently remove fault-related components during denoising, yielding signals that appear clean but lack physical interpretability [19,20]. This limitation fundamentally undermines robustness and trustworthiness in real-world diagnostic scenarios.

To overcome these limitations, we propose a denoising framework that integrates a physics-informed regression branch into a deep residual denoiser, which has been used in some studies for classification and recognition tasks [21,22]. The branch is explicitly designed to preserve and enhance physically meaningful features in vibration signals. Its design is grounded in the known dynamics of rolling element bearings and the characteristic signatures produced under fault conditions [23,24]. For instance, localized defects generate periodic impulsive forces as the flaw repeatedly contacts mating surfaces; these excitations in turn drive high-frequency structural resonances and yield amplitude-modulated responses governed by bearing kinematics [25]. Rather than treating the input as a generic time series, the physics-based branch is trained to learn and regenerate these canonical structures—transient impulses, resonance bands, and modulation sidebands—thereby steering the main denoiser toward reconstructions that respect the underlying system dynamics. Preserving such features is crucial for two reasons. First, they carry essential diagnostic information for fault localization and severity assessment; for example, the impulse repetition rate—determined jointly by fault type and shaft speed—is a direct indicator of fault location [26]. Distorting impulse shape or interval can thus cause misdiagnosis or misestimation of remaining useful life. Second, resonance frequencies and modulation patterns reflect structural properties of the mechanical system [27,28]; maintaining their integrity ensures interpretability and consistency with domain knowledge and facilitates downstream tasks such as root-cause analysis. The novelty of our proposed method compared to previous works has been shown in Table 1.

We validate the proposed approach on the widely used Case Western Reserve University (CWRU) bearing dataset, which spans multiple operating conditions and fault types and has become a de facto benchmark for fault diagnosis [29,30]. Experimental results show that our method achieves superior noise suppression relative to both traditional and state-of-the-art baselines, while substantially improving the accuracy and robustness of subsequent fault diagnosis. These findings confirm that combining low-dimensional manifold priors with deep neural architectures constitutes an effective strategy for bearing-signal denoising and diagnosis under complex operating conditions.

Our main contributions are threefold.

1. We introduce a physics-informed regression branch that encodes kinematics-consistent priors (impulse trains, resonance bands, modulation sidebands) to guide denoising.
2. We couple these priors with a residual denoiser and manifold learning to preserve diagnostically critical structures while suppressing diverse noise.
3. We provide comprehensive validation on CWRU, demonstrating consistent gains in both denoising quality and fault-diagnosis performance over strong baselines.

The remaining part of the paper is organized as follows. Section 2 details the simulation of physical characteristics in motor vibration signals and presents the architecture of the proposed PDRNet, including the residual main network and the regression branch, together with the quantitative evaluation protocol. Section 3 reports experimental setups and results, including denoising performance, periodicity score analysis, and fault-diagnosis accuracy. Section 4 concludes with a summary and discussion.

## 2. Methodology

The theoretical foundation of this work lies in the premise that vibration signals originate from the periodic kinematic interactions of gears under real-world operating conditions, thereby exhibiting distinct and physically interpretable patterns [31]. These patterns—including meshing harmonics, amplitude modulation, sideband structures, and transient impulses—are intrinsically governed by the mechanical properties and dynamic behaviors of the rotating components [32]. Consequently, it is both feasible and advantageous to exploit these inherent physical characteristics as prior knowledge to guide, regularize, and correct the learning process of denoising models. In this way, the denoising objective transcends the conventional goals of minimizing loss functions or improving signal-to-noise ratio (SNR); instead, it emphasizes the reconstruction of signals that are physically consistent and diagnostically meaningful.

To implement this concept, we propose a modified denoising architecture based on an encoder–decoder framework, augmented with a dedicated regression branch explicitly designed to capture and preserve fault-related features. The effectiveness of the proposed approach is rigorously assessed using multiple complementary metrics, including SNR, periodicity retention rate, and fault-diagnosis accuracy, thereby ensuring that both quantitative performance and functional diagnostic value are comprehensively validated.

### 2.1. Simulation of Motor Vibration Signals

Based on the theoretical foundation, we develop a comprehensive simulation framework specifically designed for motor vibration signals. In real bearing systems, clean vibration signals are fundamentally composed of two distinct components: normal operational vibrations and fault-induced vibrations. The clean signal can be mathematically expressed as

(1) $$x_{clean}\left(t\right)=x_{normal}\left(t\right)+x_{fault}\left(t\right)$$

where $x_{normal}\left(t\right)$ represents normal operational vibrations arising from periodic rotational motion, and $x_{fault}\left(t\right)$ denotes vibrations generated by bearing defects. Normal operational vibrations in rotating machinery originate from the fundamental periodic motion of the motor shaft and its mechanical components. During rotation, various mechanical phenomena including shaft rotation, mechanical imbalances, electromagnetic forces contribute to the vibration spectrum. These characteristics are modeled using sinusoidal functions.

(2) $$x_{normal}\left(t\right)=A\sin\left(2\pi \cdot f_{r}\cdot t\right)$$

where *A* represent the amplitudes of the fundamental, $f_{r}=RPM/60$ Hz represent the base rotational frequency. Bearing faults create distinctive vibration signatures through periodic contact between defective surfaces and rolling elements, which can typically be categorized into three main types: inner race faults (BPFI), outer race faults (BPFO), and rolling element faults (BSF) [25]. Each fault type exhibits unique vibration characteristics, typically manifested as periodic impulses and their corresponding frequency components in the vibration spectrum. Specifically, these fault-related frequencies—BPFI, BPFO, and BSF—are functions of the bearing geometry and rotational speed, and are commonly used as diagnostic indicators in both time and frequency domain analyses.

Each fault location’s vibration frequency is determined by bearing geometry and operating speed, enabling fault identification through frequency analysis. To accurately capture these transient impact characteristics, we employ exponentially decaying sinusoidal functions that model the physical dissipation of impact energy:

(3) $$x_{fault}\left(t\right)=A_{fault}\cdot e^{-\lambda (t\;\mathrm{mod}\;T)}\cdot \sin\left(2\pi \cdot f_{fault}\cdot t\right)$$

where indexes different fault types, represents harmonic order, $A_{fault}$ and $f_{fault}$ denotes the fault severity amplitude and frequency respectively, λ is the exponential decay constant modeling impact dissipation, $T=1/f_{fault}$ represents the period. The exponential decay term $e^{-\lambda (t\;\mathrm{mod}\;T)}$ simulates the transient nature of bearing impacts, where each impact rapidly dissipates before the subsequent occurrence. In practical bearing condition monitoring applications, measured vibration signals inevitably consist of clean signal components contaminated by various noise sources. The complete measured signal can be expressed as

(4) $$x_{real}\left(t\right)=x_{clean}\left(t\right)+x_{noise}\left(t\right)$$

The noise component $x_{real}\left(t\right)$ encompasses multiple sources of interference commonly encountered in industrial environments: measurement noise from sensors and data acquisition systems introduces electronic artifacts, environmental interference from adjacent machinery and structural vibrations creates background contamination, and process variations in motor loading and operating conditions contribute to stochastic fluctuations. To model these diverse noise sources, additive white Gaussian noise (AWGN) is employed:

(5) $$x_{noise}\left(t\right)∼N\left(0,\sigma^{2}\right)$$

### 2.2. Proposed Architecture

**Figure 1:**
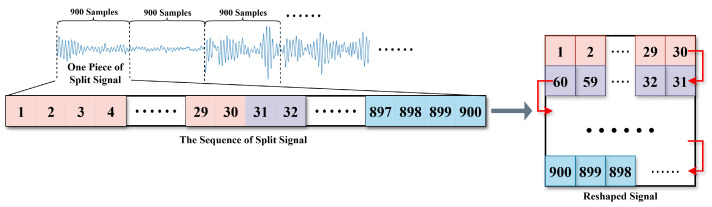
The schematic diagram of data preproccessing.

**Figure 2:**
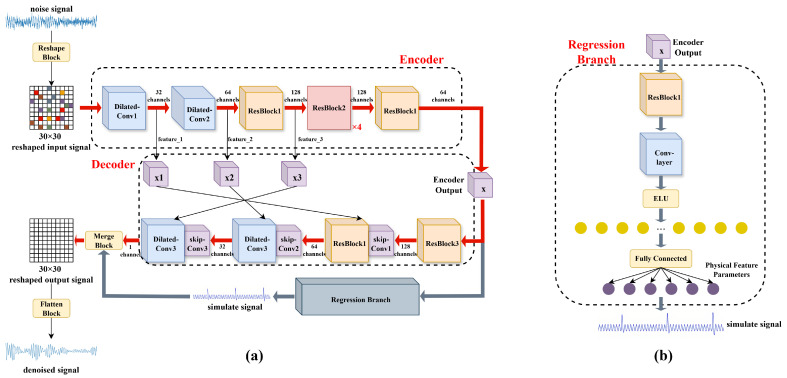
PDRNet. (**a**) Description of the overall model structure consists of an encoder, a decoder and a regression branch. (**b**) The structural details of the regression branch.

We propose a novel signal denoising framework that augments a conventional encoder–decoder architecture with an auxiliary regression branch, thereby enhancing denoising performance through explicit clean-signal simulation. The key components of the proposed approach are summarized as follows:

1. **Data Pre-Processing.** The raw dataset is segmented into non-overlapping windows of 900 sampling points per data sample. To exploit spatial convolution operations and improve contextual understanding, the one-dimensional time-series signal is reshaped into a two-dimensional representation. Specifically, each 900-point sequence is rearranged into a serpentine pattern, forming a 30×30 matrix in which temporal adjacency is preserved, as illustrated in Figure 1. This data preprocessing method has been proven to have a significant effect on TEM signals [33]. This mapping strategy maintains the temporal continuity of the original signal while enabling convolutional layers to capture both local and global contextual dependencies. Following the forward pass, the resulting 30×30 feature map is flattened back into the one-dimensional format.
2. **Dilated Convolutions and Residual Learning.** To enlarge the receptive field without increasing the number of parameters or reducing spatial resolution, dilated convolutions are employed at both the encoder’s input stage and the decoder’s output stage [34], thereby capturing multi-scale contextual information efficiently (Figure 2a). In parallel, we adopt a deep residual learning framework comprising three types of residual blocks, which facilitate easier training and improved performance by allowing gradients to flow more effectively through the network, mitigating the vanishing gradient problem [35]. Each block integrates three convolutional layers followed by a residual connection. This design facilitates the modeling of complex noise structures and signal characteristics that require multi-level abstraction, ultimately improving the network’s representational capacity in challenging denoising scenarios.
3. **Multi-Level Skip Connections.** To preserve fine-grained information during reconstruction, skip connections are established at three hierarchical levels [36]. Specifically, intermediate features are extracted after Dilated-Conv1 (32 channels), Dilated-Conv2 (64 channels), and the first ResBlockV1 (128 channels) in the encoder. These features are progressively fused in the decoder through concatenation operations, followed by 1×1 convolutional layers (skip-conv) that adaptively integrate multi-resolution information. By retaining high-resolution details lost in conventional encoder–decoder structures, this design substantially improves reconstruction fidelity.
4. **Regression Branch Innovation.** An auxiliary regression branch is introduced to explicitly model the underlying clean-signal characteristics. Unlike conventional methods that rely solely on reconstruction loss between noisy input and denoised output, the regression branch extracts intermediate encoder features and processes them to generate a simulated clean-signal representation. The branch consists of a residual block, a convolutional layer with ELU activation, and a fully connected network that outputs physical feature parameters (Figure 2b). This additional pathway provides strong physical priors, ensuring that the denoising process respects the inherent dynamics of the original signal.

### 2.3. Performance Evaluation Methodology

To comprehensively evaluate the effectiveness of the proposed physical feature prior–guided denoising model, we adopt a multi-dimensional assessment framework that encompasses signal quality indicators, physical feature preservation analysis, and practical diagnostic performance.

#### 2.3.1. Signal Quality Assessment

The primary metric for evaluating denoising performance is the Signal-to-Noise Ratio (SNR), which quantifies the improvement in signal clarity achieved after denoising. It is defined as

(6) $$SNR=10\log_{10}\left(\frac{P_{signal}}{P_{noise}}\right)$$

where $P_{signal}$ denotes the power of the clean signal, and $P_{noise}$ represents the power of the residual noise.

#### 2.3.2. Physical Feature Preservation Assessment

In addition to signal quality, it is critical to evaluate how effectively the denoising process preserves the intrinsic physical characteristics of bearing vibration signals. To this end, we adopt a comprehensive periodicity analysis framework that integrates multiple complementary indicators:

Autocorrelation Peak Analysis. This metric measures the strength of periodic components by computing the maximum normalized autocorrelation coefficient:

(7) $$A_{score}=\max\left(\frac{R_{xx}\left(\tau \right)}{R_{xx}\left(0\right)}\right)$$

where $R_{xx}\left(\tau \right)$ is the autocorrelation function at lag τ.

Spectrum Peak Ratio. This indicator quantifies the dominance of the fundamental frequency component relative to the average spectral energy:

(8) $$SP_{score}=\frac{\left|X(f_{main})\right|}{\frac{1}{N}\sum_{k=1}^{N/2}\left|X(f_{k})\right|}$$

where $\left|X(f_{main})\right|$ denotes the amplitude at the main frequency, and *N* is the number of frequency bins.

Spectral Entropy. This metric evaluates the concentration of spectral energy, with lower values indicating stronger periodicity:

(9) $$SE_{score}=-\sum_{k=1}^{N/2}P\left(f_{k}\right)\log_{2}P\left(f_{k}\right)$$

where $P(f_{k})$ is the normalized power spectral density.

Periodicity Consistency. This indicator measures waveform similarity across consecutive periods using Pearson correlation coefficients:

(10) $$PC_{score}=\frac{1}{M-1}\sum_{i=1}^{M-1}\rho \left(T_{i},T_{i+1}\right)$$

where *M* is the number of complete signal periods, $T_{i}$ and $T_{i+1}$ denote adjacent periods, and $\rho (T_{i},T_{i+1})$ is their Pearson correlation coefficient.

Finally, an Overall Periodicity Score is computed as a weighted combination of the above indicators:

(11) $$Periodicity-Score=0.4\times A_{score}+0.3\times SP_{score}+0.2\times SE_{score}+0.1\times PC_{score}$$

This comprehensive evaluation system ensures that the denoising process not only suppresses noise but also preserves the inherent periodic features that are fundamental for accurate bearing fault diagnosis.

#### 2.3.3. Evaluation of Diagnostic Performance

To evaluate the practical utility of denoised signals, we primarily use the F1 score to assess fault detection performance. The F1 score is particularly suitable for bearing fault diagnosis due to its robustness against class imbalance, which is common in industrial datasets where normal operating conditions typically outnumber fault conditions. For each fault class, the F1 score is calculated as

(12) $$F1\_score=\frac{2\cdot precision\cdot recall}{precision+recall}$$

where

(13) $$\left\{\begin{array}{c} precision=\frac{TP}{TP+FP}\\ recall=\frac{TP}{TP+FN} \end{array}\right.$$

and TP, FP, FN represent true positives, false positives, and false negatives, respectively.

## 3. Results

### 3.1. Dataset and Experiment Setup

The motor vibration data used in this study are sourced from the well-established CWRU Bearing Dataset. Data were collected using accelerometers mounted at the drive end and the fan end of the motor, with sampling frequencies of 12 kHz and 48 kHz, respectively. In this work, we specifically utilize the drive-end signals sampled at 48 kHz. Following the segmentation strategy described in Section 2.2, a total of 25,798 samples were obtained and subsequently divided into training and validation sets with a ratio of 9:1 for the main denoising experiments. When evaluating fault diagnosis performance, the dataset contained vibration signals corresponding to four health conditions: normal, inner race fault, outer race fault, and ball fault, which were approximately equally represented in the diagnostic dataset. After deleting and padding zeros for the data that is not a multiple of signal length we set, which is 900, the total number of samples for each class used in the diagnostic experiments is: 6693 samples for ball faults, 6490 samples for inner race faults, 8740 samples for outer race faults and 3886 samples for normal. Each fault type was induced with three defect sizes (0.007, 0.014, and 0.021 inches in diameter), and vibration data were recorded under motor loads of 0, 1, 2, and 3 horsepower. Based on different operating conditions and defect sizes, the dataset is categorized into ten distinct classes, as summarized in Table 2.

### 3.2. Experiments

To assess the generalization capability of the proposed PDRNet in motor vibration signal denoising tasks, we design a series of experiments targeting different influencing factors. Specifically, these experiments verify (i) the superior performance of PDRNet compared with existing denoising models, (ii) the contribution of the physics-informed regression branch to the model’s denoising ability, and (iii) the critical role of the regression branch in preserving the physical features of the denoised signals.

To better quantify denoising performance, we construct a composite loss function comprising the mean squared error (MSE) from the decoder, denoted as $Loss_{decoder}$, and that from the regression branch, denoted as $Loss_{exp}$. The MSE is defined as

(14) $$MSE=\frac{1}{N}\sum_{i=1}^{N}{\left(y_{i}-{\hat{y}}_{i}\right)}^{2}$$

where $y_{i}$ denotes the original clean signal, ${\hat{y}}_{i}$ represents the denoised signal, and *N* is the total number of samples. The overall loss is then obtained as a weighted combination:

(15) $$Loss_{total}=\alpha \cdot Loss_{decoder}+\beta \cdot Loss_{exp}$$

where α and β are weighting coefficients.

The experiments are conducted on a 64-bit Windows 11 operating system, using Python 3.7.1 as the programming language and PyTorch 1.10.0+cu113 as the deep learning framework. Visual Studio Code 1.89.1 serves as the integrated development environment (IDE), while the hardware platform is a laptop equipped with an NVIDIA GeForce RTX 3060 GPU. After multiple rounds of training and evaluation, the final set of hyperparameters is determined, as summarized in Table 3.

To achieve optimal model performance, we adopted an empirical tuning strategy. Initial hyperparameter values were selected based on commonly used configurations in deep learning for signal processing, and subsequently refined by monitoring the model’s performance on the validation set. The number of training epochs was set to 200, as we observed that the validation loss and performance metrics plateaued around this value. In particular, the weighting coefficients for the loss terms were set to α=0.7 and β=0.3. This configuration emphasizes the contribution of the regression branch, which captures essential physical features, while still preserving the complex structural components of real-world signals through the encoder–decoder pathway.

Under the full dataset and standard training settings, the total training time for PDRNet was approximately 5 h, with each epoch requiring around 90 s on average.

It’s worth noting that we adopted Cosine Annealing learning rate scheduler to ensure stable and efficient convergence during training [37]. This strategy gradually reduces the learning rate following a cosine curve from an initial maximum value to a minimum, enabling smoother convergence and helping the model escape shallow local minima in the early stages. Compared to traditional step decay or exponential decay, cosine annealing provides non-monotonic and continuous decay, which often improves convergence stability and prevents premature stagnation [38]. Step decay reduces the learning rate at fixed intervals, which can cause abrupt changes in training dynamics. Exponential decay decreases the learning rate continuously but lacks the restart capability offered by cosine annealing. In our experiments, cosine annealing was proved to yield more stable training and better generalization across various noise levels without requiring extensive manual tuning of hyperparameters.

#### 3.2.1. Denoising Capability Assessment

Based on the results summarized in Table 4, PDRNet consistently outperforms all competing models across different noise intensity levels (−3 dB, 0 dB, and 3 dB). The improvement is particularly pronounced when the regression branch (Reg-branch) is incorporated, which markedly enhances the Signal-to-Noise Ratio (SNR). Specifically, PDRNet achieves an SNR of 7.817 at −3 dB, 9.401 at 0 dB, and 12.51 at 3 dB, all of which clearly surpass the corresponding values of other models under identical conditions. Even without the regression branch, PDRNet still demonstrates competitive performance, although the SNR values are reduced compared with the Reg-branch configuration.

In contrast, DnCNN, while effective in mitigating noise, fails to reach the performance level of PDRNet, especially when the regression branch is applied. FFDNet exhibits relatively stable performance across different noise levels but remains inferior to PDRNet. U_Net struggles under low signal-to-noise ratio conditions, resulting in unsatisfactory denoising outcomes. RND maintains stable performance across varying noise intensities, yet it also falls short of PDRNet in overall effectiveness. Collectively, these results confirm that PDRNet, particularly with the regression branch, represents the most effective solution for denoising motor vibration signals under diverse noise conditions.

**Figure 3:**
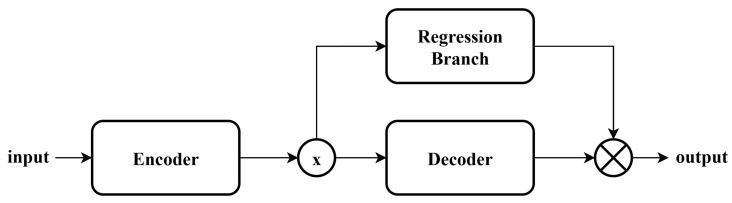
The schematic diagram of modified architecture. The symbol ⊗ represents the merge block.

To further examine the role of the regression branch, we incorporated it into encoder–decoder architectures based on DnCNN and FFDNet, as shown in Figure 3. Interestingly, the results show that the regression branch actually degraded their performance. For example, at 0 dB, DnCNN achieved an SNR of 8.939 without the regression branch but only 8.768 with it, while FFDNet produced an SNR of 8.961 without the branch compared with 8.940 when it was added. This observation suggests that the regression branch, grounded in physical feature priors, is not universally compatible with all model structures. In these modified networks, the encoding process appears unable to adequately capture the underlying data features, thereby preventing the regression branch from effectively extracting the relevant physical characteristics.

**Figure 4:**
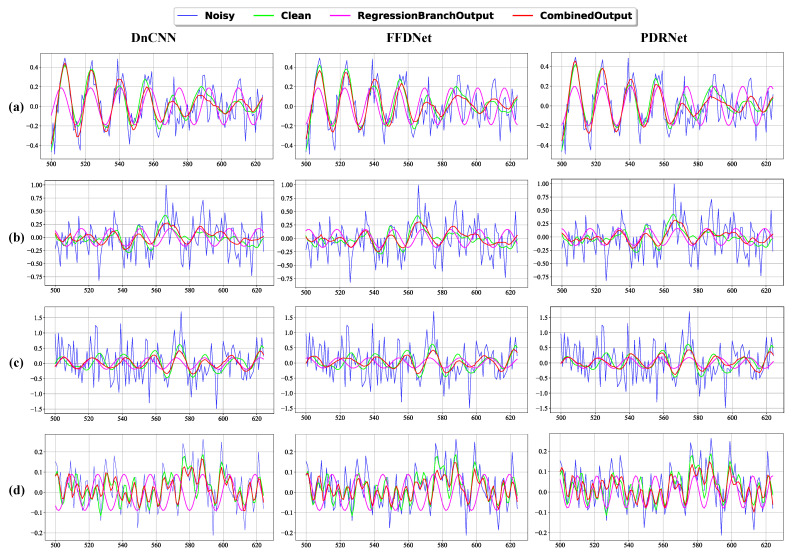
The visualized results of different denoising models, including DnCNN, FFDNet and PDRNet when motor operating under different fault conditions. Row (**a**), row (**b**), row (**c**), row (**d**) represent Ball fault, InnerRace fault, OuterRace fault and Normal condition respectively. The signals shown in the figure are data collected from our divided dataset, specifically at the 500–625 sampling points. The x-axis represents the sampling points, while the y-axis represents the signal amplitude.

Figure 4 provides a visual comparison of the denoising results across different working conditions obtained using different denoising models, further illustrating the advantages of PDRNet with the regression branch over the benchmark methods.

#### 3.2.2. Periodic Assessment of Noise-Reduced Signals

To objectively evaluate the ability of different denoising models to restore the underlying rotational regularity of motor vibration signals, we adopt the periodicity score as a key performance metric. This score ranges from 0 to 1, with higher values indicating stronger and more coherent periodic structures within the signal. By integrating features from both the time and frequency domains, the periodicity score provides a comprehensive measure of signal integrity.

**Figure 5:**
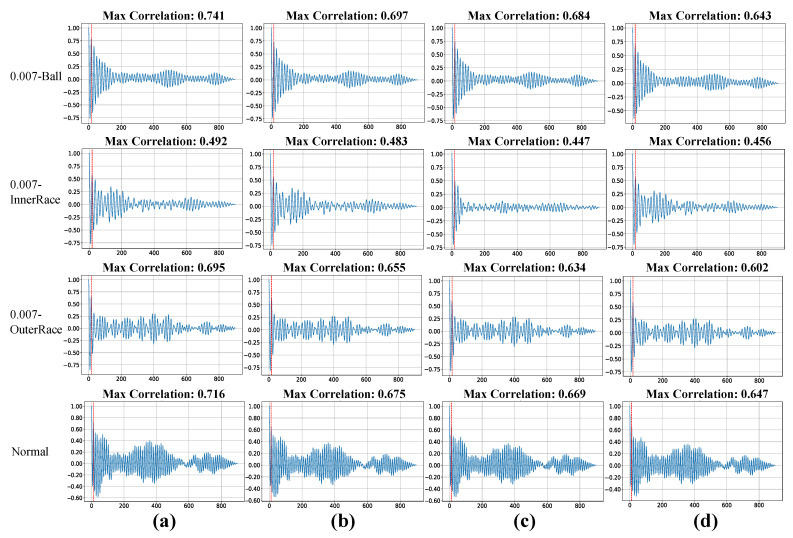
The autocorrelation function graph of the signal through different denoising model. Four rows represent Ball fault, Inner Race fault, Outer Race fault and Normal condition respectively. Column (**a**) represent clean signal, while column (**b**–**d**) represent noise signal processed by denoising model PDRNet, RND and DnCNN respectively. The red dotted line indicates the point where the signal reaches the peak of autocorrelation when the period is 18 or 19.

To illustrate the relationship between periodicity and autocorrelation peaks, representative autocorrelation functions from four operating conditions (Ball fault, Inner Race fault, Outer Race fault, and Normal condition) are presented in Figure 5. The results clearly show that signals processed by the proposed method exhibit substantially higher autocorrelation peak values in all working cinditions compared with those produced by competing models. A higher peak reflects better preservation and enhancement of the intrinsic periodic structure, with our approach achieving a peak value of 0.694 in Ball fault condition. In contrast, the other models yield lower peak values, suggesting that residual noise or distortion persists in their outputs, thereby disrupting periodicity and weakening alignment across signal cycles. This finding underscores the superior capability of our method in recovering the cyclic patterns of vibration signals, which is essential for reliable condition monitoring and accurate fault diagnosis.

**Figure 6:**
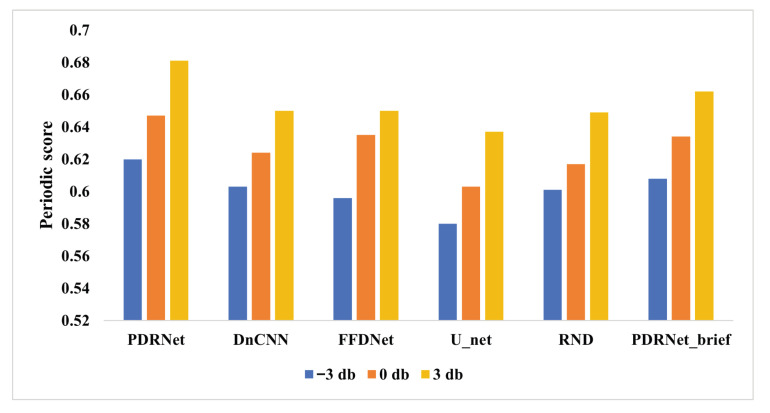
Periodic scores of different models under different SNR. PDRNet_brief is PDRNet without regression branch.

To further assess performance under varying noise intensities, we compute the periodicity score for signals processed by each model, as summarized in Figure 6. The results consistently highlight the significant advantage of PDRNet in preserving the core characteristics of motor vibration signals. For instance, at a noise level of 3 dB, our model achieves a periodicity score of 0.681, compared with 0.650 for DnCNN, 0.650 for FFDNet, 0.637 for U_Net, and 0.649 for RND. Importantly, the physical feature regression branch plays a pivotal role in maintaining periodic structure, as evidenced by the reduced score of 0.662 for PDRNet_brief, the variant without this branch. This result emphasizes the necessity of incorporating physical feature priors to effectively preserve the periodic structure and overall integrity of motor vibration signals, thereby demonstrating the substantial benefits of the proposed approach for real-world industrial applications.

#### 3.2.3. Bearing Fault Diagnosis

To examine whether the proposed denoising model improves bearing fault detection under realistic working conditions, we used three widely used diagnostic models: WDCNN [39], CNN-LSTM [40], and ResNet [41] to evaluate the classification performance of signals processed by different denoising methods. The F1-scores of signals with varying noise levels under these diagnostic frameworks are reported in Table 5. Overall, PDRNet consistently outperforms competing denoising models across multiple noise levels and diagnostic architectures.

When WDCNN was used as the diagnostic model, PDRNet achieved accuracies of 86.87% at −3 dB, 94.69% at 0 dB, and 97.33% at 3 dB. With CNN-LSTM, the corresponding results were 69.93%, 87.14%, and 94.54%. Using ResNet, PDRNet reached 87.06% at −3 dB, 94.46% at 0 dB, and 97.13% at 3 dB. Notably, when ResNet was used as the diagnostic model and the input contained 0 dB noise, DnCNN without the regression branch slightly outperformed PDRNet, achieving an accuracy of 94.54%. Furthermore, as shown in Table 5, the denoising performance of DnCNN and FFDNet actually deteriorated after introducing the regression branch, and the same trend was observed in their diagnostic results. This finding indicates that the regression branch requires input signals containing meaningful feature content to accurately capture and reconstruct the physical characteristics of vibration signals.

Among the three diagnostic models, ResNet demonstrated the best overall performance, achieving 97.13% accuracy at 3 dB and maintaining robust fault detection even under severe noise conditions, with 87.06% accuracy at −3 dB. By contrast, CNN-LSTM exhibited the weakest diagnostic capability, particularly in the presence of substantial noise. For instance, when combined with PDRNet for signals at −3 dB, the highest accuracy achieved was only 69.93%.

## 4. Conclusions

In conclusion, this study presents a novel denoising framework that seamlessly integrates physical feature priors, offering a robust approach to noise suppression while preserving the integrity of diagnostically critical signal characteristics. The framework incorporates a regression branch constructed upon convolutional and residual neural networks, which guides the denoising process to ensure that key physical features are retained while noise is effectively eliminated. Experimental evaluations demonstrate that the proposed method not only outperforms conventional denoising techniques in terms of accuracy but also substantially enhances the performance of bearing fault diagnosis systems. By leveraging physical feature priors, the model transcends the traditional objective of minimizing loss functions, enabling it to capture the inherent physical properties of bearing signals. Specifically, the proposed PDRNet, which integrates the physical feature regression branch and residual network, achieves notable improvements in signal-to-noise ratio (SNR), with gains of 7.817, 9.401, and 12.51 dB at −3 dB, 0 dB, and 3 dB noise levels, respectively. These results consistently surpass those of established denoising networks such as DnCNN, FFDNet, U_Net, and RND. Among these, RND demonstrates relatively close performance, with SNR values of 7.398, 9.305, and 12.432 dB under the same noise conditions. However, when the regression branch is removed from PDRNet, the denoising performance declines, yielding SNRs of only 7.032, 9.176, and 12.257 dB.

In addition, periodicity analysis further highlights the role of the regression branch. Under a 3 dB noise level, PDRNet with the regression branch achieves a periodicity score of 0.681, whereas its counterpart without the branch yields a slightly lower score of 0.662. This difference underscores the critical importance of incorporating physical feature priors in preserving the intrinsic cyclic structures of bearing signals. Overall, the proposed framework provides a reliable and effective solution for denoising bearing signals in industrial environments characterized by high variability and complex noise. Beyond improving denoising accuracy, this advancement holds significant potential for enhancing predictive maintenance strategies, improving condition monitoring reliability, and deepening the understanding of machine health, thereby contributing to more efficient and proactive industrial operations.

Nevertheless, this study also has certain limitations. The proposed model has been validated only on the CWRU benchmark dataset, which may not fully capture the complexity of real-world industrial environments. Moreover, the computational cost associated with deep model training also limits its direct deployment in resource-constrained embedded systems. Future research will focus on expanding the evaluation to field-collected vibration datasets under various conditions, introducing domain adaptation to enhance generalization, and exploring lightweight model designs for real-time industrial applications and deployment on edge devices. Furthermore, it would be beneficial to integrate multimodal sensory data, such as acoustic, current, and temperature signals, to further improve diagnostic robustness and interpretability.

## Figures and Tables

**Table 1:** Comparison between existing vibration signal denoising methods and the proposed PDRNet.

Method	Physics-Driven	Residual Net	Signal Feature Preservation
Traditional methods	✗	✗	✗
DnCNN	✗	✗	Partial
FFDNet	✗	✗	Partial
RND	✗	✓	Partial
PDRNet (our model)	✓	✓	✓

**Table 2:** The composition of our dataset, in which the data is derived from the 48k sampling rate data in the CWRU dataset. Each class number corresponds to a unique fault type and severity level used in this study.

Class No.	Defect Size (Inch)	Fault Type	Number
0	0.007	Ball	2231
1	0.007	Inner-race	2161
2	0.007	Outer-race	2913
3	0.014	Ball	2233
4	0.014	Inner-race	2163
5	0.014	Outer-race	2913
6	0.021	Ball	2229
7	0.021	Inner-race	2166
8	0.021	Outer-race	2914
9	0	Normal	3886

**Table 3:** Model hyperparameter configuration.

Hyperparameter	Value
Batch size	128
Number of epochs	200
Learning rate scheduler	Cosine Annealing
Optimizer	Adam
Initial learning rate	0.001
α	0.7
β	0.3

**Table 4:** The noise reduction capabilities of different models for signals with different noise intensities, with or without the regression branch.

Model	If Reg-Branch	SNR (dB)		
		−3 dB	0 dB	3 dB
PDRNet	✓	**7.817**	**9.401**	**12.51**
	✗	7.032	9.176	12.257
DnCNN	✓	6.724	8.768	11.48
	✗	6.81	8.939	12.081
FFDNet	✓	7.136	8.94	11.903
	✗	7.463	8.961	11.97
U_net	✓	6.38	8.093	11.02
	✗	6.491	8.354	11.337
RND	✓	7.549	8.98	12.491
	✗	7.398	9.305	12.432

**Note:** The bold numbers represent the best performance for each SNR level. The meaning is consistent across all tables.

**Table 5:** Under different fault diagnosis models, a comparison of the bearing fault diagnosis performances of the signals after going through different denoising models.

Model		WDCNN			CNN-LSTM			ResNet		
SNR (dB)		−3	0	3	−3	0	3	−3	0	3
Denoising Model	If Reg-Branch	F1-Score (%)								
PDRNet	✓	**86.87 ± 0.22**	**94.69 ± 0.44**	**97.33 ± 0.37**	**69.93 ± 0.36**	**87.14 ± 0.11**	**94.54 ± 0.43**	**87.06 ± 0.15**	94.46 ± 0.26	**97.63 ± 0.17**
	✗	84.11 ± 0.22	89.35 ± 0.23	93.79 ± 0.41	62.47 ± 0.35	85.68 ± 0.47	90.53 ± 0.24	86.92 ± 0.47	92.24 ± 0.19	95.16 ± 0.25
DnCNN	✓	77.40 ± 0.28	92.84 ± 0.37	93.65 ± 0.11	50.72 ± 0.42	85.57 ± 0.34	90.99 ± 0.27	79.19 ± 0.25	93.31 ± 0.13	95.22 ± 0.49
	✗	77.22 ± 0.14	93.18 ± 0.17	96.82 ± 0.38	51.49 ± 0.46	86.90 ± 0.32	93.68 ± 0.49	81.94 ± 0.12	**94.54 ± 0.22**	96.47 ± 0.11
FFDNet	✓	77.76 ± 0.44	92.91 ± 0.18	94.54 ± 0.20	50.13 ± 0.37	68.26 ± 0.05	83.87 ± 0.46	76.84 ± 0.14	90.70 ± 0.39	96.33 ± 0.13
	✗	78.54 ± 0.18	92.95 ± 0.43	96.44 ± 0.15	52.05 ± 0.35	69.53 ± 0.32	84.56 ± 0.27	77.41 ± 0.19	92.13 ± 0.21	96.40 ± 0.28
U_net	✓	72.46 ± 0.23	90.28 ± 0.34	95.31 ± 0.13	47.38 ± 0.47	59.51 ± 0.43	89.65 ± 0.27	75.40 ± 0.39	89.50 ± 0.19	95.58 ± 0.19
	✗	73.60 ± 0.16	91.42 ± 0.38	96.37 ± 0.12	52.95 ± 0.33	64.50 ± 0.20	92.69 ± 0.46	77.18 ± 0.49	91.86 ± 0.31	96.42 ± 0.28
RND	✓	85.66 ± 0.14	90.20 ± 0.28	96.98 ± 0.44	53.74 ± 0.43	64.10 ± 0.31	94.27 ± 0.37	86.13 ± 0.27	91.24 ± 0.10	96.93 ± 0.38
	✗	85.67 ± 0.22	89.83 ± 0.19	94.28 ± 0.38	51.12 ± 0.36	62.25 ± 0.45	93.73 ± 0.23	83.49 ± 0.22	89.62 ± 0.27	94.41 ± 0.11

## Data Availability

Data and source code used in the paper can be accessed by contacting the authors.
